# High-throughput primer design by scoring in piecewise logistic model for multiple polymerase chain reaction variants

**DOI:** 10.1038/s41598-022-25561-z

**Published:** 2022-12-07

**Authors:** Huaping Zeng, Kexin Chen, Chouxian Ma, Biyin Zhu, Jun Chuan, Shuan Zhang, Lin Tang, Ting Yang, Zhaohui Sun, Xingkun Yang, Yu Wang

**Affiliations:** 1 School of Life Sciences and Technology, Tongji University, Shanghai, 200092 China; 2 GeneTalks Biotech Co., Ltd., Changsha, 410000 Hunan China; 3 Affliated Foshan Maternity & Child Healthcare Hospital, Southern Medical University, Foshan, 528000 Guangdong China

**Keywords:** Computational models, Bioinformatics, High-throughput screening

## Abstract

Polymerase chain reaction (PCR) variants requiring specific primer types are widely used in various PCR experiments, including generic PCR, inverse PCR, anchored PCR, and ARMS PCR. Few tools can be adapted for multiple PCR variants, and many tools select primers by filtration based on the given parameters, which result in frequent design failures. Here we introduce PrimerScore2, a robust high-throughput primer design tool that can design primers in one click for multiple PCR variants. It scores primers using a piecewise logistic model and the highest-scored primers are selected avoiding the issue of design failure and the necessity to loosen parameters to redesign, and it creatively evaluates specificity by predicting the efficiencies of all target/non-target products. To assess the prediction accuracy of the scores and efficiencies, two next generation sequencing (NGS) libraries were constructed—a 12-plex and a 57-plex—and the results showed that 17 out of 19 (89.5%) low-scoring pairs had a poor depth, 18 out of 19 (94.7%) high-scoring pairs had a high depth, and the depth ratios of the products were linearly correlated with the predicted efficiencies with a slope of 1.025 and a coefficient of determination (R^2^) 0.935. 116-plex and 114-plex anchored PCR panels designed by PrimerScore2 were applied to 26 maternal plasma samples with male fetuses, the results showed that the predicted fetal DNA fractions were concordant with fractions measured in gold standard method (Y fractions). PrimerScore2 was also used to design 77 monoplex Sanger sequencing primers, the sequencing results indicated that all the primers were effective.

## Introduction

Multiple polymerase chain reaction (PCR) variants are used in numerous DNA-based molecular experiments. As well as the well-known generic PCR which uses primers in face-to-face orientation, inverse PCR has been developed to amplify cyclized DNA molecules^1^ and needs primers in back-to-back orientation, while anchored PCR using primers with a unidirectional orientation is widely used to detect gene or virus fusions with an unknown partner^2^. Since the fusion position is usually unknown, it is necessary to design several primers fully and evenly covering the entire gene or virus region. And these PCR variants are usually applied to multiplex PCR in next-generation sequencing (NGS), due to its high efficiency and low cost. Therefore, a high-throughput primer design tool suitable for multiple PCR variants is in demand. Regardless of the PCR variant used, the main factors affecting the performance of primers remain consistent, including melting temperature (Tm), GC content, product size, specificity, self-complementarity, and common SNPs covered by the primers^3–5^ and cross-dimers in multiplex panels. This situation provides a basis for the development of a universal primer design tool.

Currently, the most widely used primer design tools, such as Primer3^6^, Primer Premier (http://www.premierbiosoft.com/primerdesign/index.html), OLIGO (Molecular Biology Insights, Inc., Colorado Springs, CO, USA), and PrimerBlast^7^, cannot design primers in bulk; researchers need to manually select one primer pair by balancing various features. Many high-throughput design tools have been published recently, such as Ultiplex^8^, NGS-PrimerPlex^9^, Oli2go^10^, MPD^11^, PrimerMapper^12^, MPprimer^13^, MFEprimer^14,15^, and BatchPrimer3^16^, none of them can design both inverse and anchored primers. They also either lack evaluation of some features, such as common SNPs and specificity, or consist of several scattered packages, which are complicated to use. Moreover, most of them select primers by filtration according to the given parameters and at the end of design task they may tell you design failure and you should loosen the parameters to start design again, and after you loosened parameters painstakingly and started again, it may still design failed, this overall process is time-consuming and frustrating. In addition, the number of current primer design tools is huge^17^, but many of them are usually specifically designed for a particular PCR variant and are not robust enough due to their inadequate development and optimization, it is tedious and difficult for researchers to test and select a suitable tool from the huge pool of tools. It is therefore important to develop a versatile and robust high-throughput primer design tool which produces no design failures and is applicable to several variants of PCR.

In this study, we developed a high-throughput primer design tool that addresses almost all of the above problems. (1) It can design multiple types of primers with any orientation, including generic PCR primers with a face-to-face orientation, anchored PCR primers with a unidirectional orientation, and inverse PCR primers with a back-to-back orientation, and can design evenly fully covered primers over the entire template. (2) It scores each feature using a piecewise logistic model and calculates the weighted sum of all feature scores to produce the final scores. The highest-scored primer pairs are selected, so the problem of design failure and the need to constantly loosen parameters and redesign are avoided. (3) It is a user-friendly, fully-automatically design tool that needs only one click to finish the design task and output three user-defined highest-scored primer pairs. (4) It examines various features of the candidate primers, including checks of common SNPs and specificity, as well as cross-dimers when designing multiplex panels. (5) It also predicts the amplification efficiency of each non-target product using a piecewise logistic model, so that the specificity can be evaluated precisely.

## Methodology

### Design tasks

**Figure 1:**
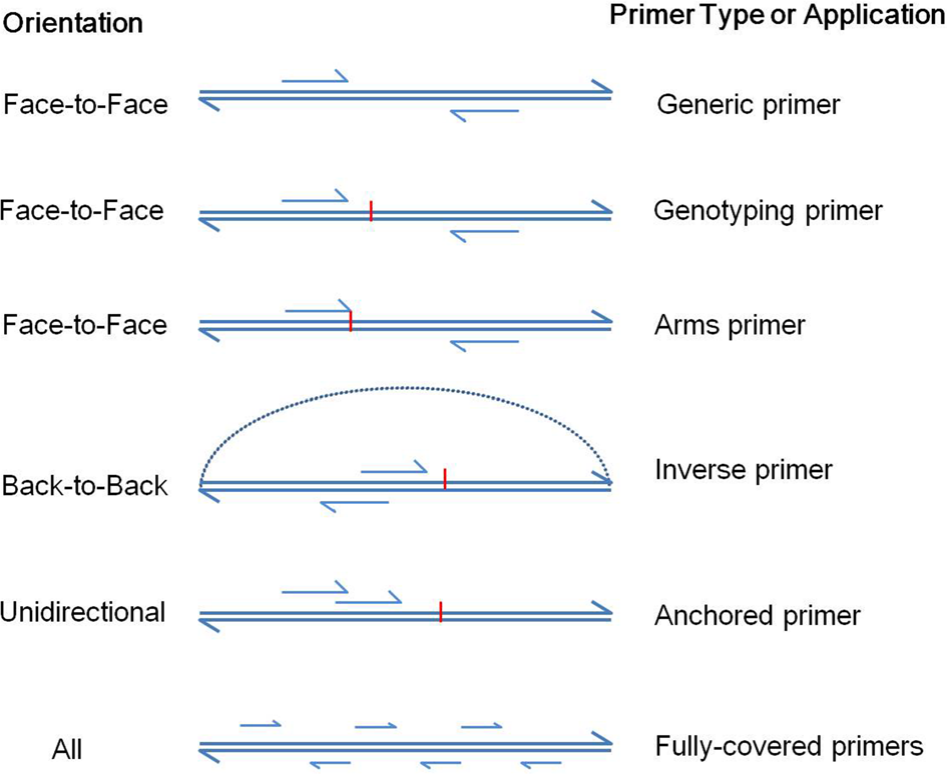
Orientations and types of primers that can be designed using PrimerScore2. The red vertical bars indicate targets (SNPs, insertions, deletions, or targeted regions).

PrimerScore2 can design primers with any orientation, such as face-to-face, back-to-back, or unidirectional orientation. It can carry out the following tasks (Fig. 1).

#### Design generic primers (face-to-face)

PrimerScore2 can design generic primers with a face-to-face orientation on the template regions. By default, the three highest-scored pairs will be selected for each region.

#### Design genotyping primers (face-to-face)

PrimerScore2 can design primers with a face-to-face orientation on the flanks of targets (SNPs, insertions, deletions, or targeted regions). ARMS primers are supportive. The distance from forward primer to target can be user defined.

#### Design inverse primers (back-to-back)

PrimerScore2 can design primers with a back-to-back orientation for inverse PCR to amplify circular DNA, such as plasmid DNA or synthetic circular DNAs like cSMART^1^. The distance between forward primer and reverse primer can be user defined.

#### Design anchored primers (unidirectional)

PrimerScore2 can design primers with a unidirectional orientation for anchored PCR. The distance between the first primer and the second primer can be user defined.

#### Design fully-covered primers

PrimerScore2 can design a suite of primers evenly covering an entire template, to detect the position of unknown inserts, deletions, or fusions of genes. The distance between two adjacent primers or the number of primers on the whole region can be user defined.

#### Evaluate pre-designed primers

PrimerScore2 can evaluate pre-designed primers. As the design tasks above, it evaluates and scores all features of the primers.

### General workflow

**Figure 2:**
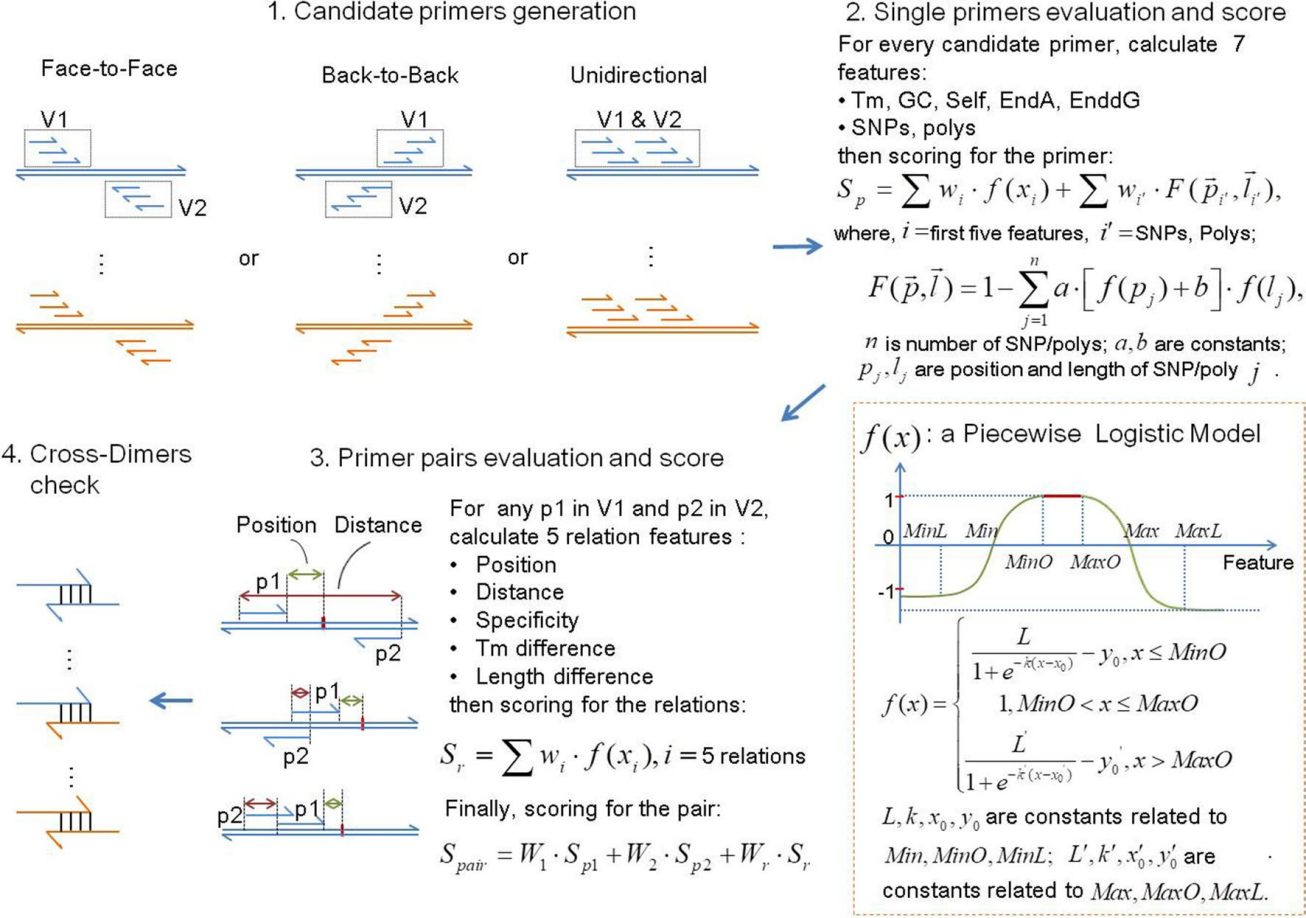
Overview of PrimerScore2. In Step 1, candidate primers are generated according to the pre-defined orientation and distance, V1 is the Primer1 set and V2 is the Primer2 set. In Step 2 seven features of each candidate primer are calculated: Tm, GC, self-complementarity (Self), ‘A’s of the 3′ end (EndA), the free energy of the 3′ end (EnddG), common SNPs covered (SNPs), and tandem repeats (polys). These features are then scored using a piecewise logistic model, and the weighted sum of these scores is the primer’s score. Step 3 involves the calculation and scoring of five relation features of each candidate pair containing a Primer1 from V1 and a Primer2 from V2 and calculates the weighted sum of these scores as the relation’s score. The final score of the pair is the weighted sum of the scores of Primer1, Primer2, and the relation. Each feature score is calculated using a piecewise logistic model with the parameters $MinO$, $MaxO$, $Min$, $Max$, $MinL$, and $MaxL$, which are the minimum and maximum of the optimal range (score equals 1, the full score), the minimum and maximum (score equals 0), and the limited minimum and maximum (score reaches the minimum value, usually a negative value), respectively.

The PrimerScore2 workflow includes four main steps (Fig. 2).

1. Generation of candidate primers by “walking” along templates.
2. Evaluation and scoring of each single candidate primer. The features of every candidate primer are calculated and then scored separately using a piecewise logistic model, and a weighted sum of the scores of all the features is calculated as the score of the primer.
3. Pair evaluation and scoring. For every candidate primer pair in which Primer1 and Primer2 meet a pre-defined orientation and distance, the relation features between Primer1 and Primer2 are calculated and scored separately using a piecewise logistic model, and then a weighted sum of scores of all the relation features is calculated as the relation’s score. The weighted sum of Primer1’s score, Primer2’s score and the relation’s score is the final score of the primer pair.
4. Check the cross-dimers among the primers of all templates when designing multiplex primers. Three highest-scoring primer pairs of each template are checked.

Below, we provide details of the implementation of each step.

#### Generation of candidate primers

Candidate primers are generated by “walking” along the interested region (user defined or automatically calculated) using a given step size, with the primer length varied from the minimum to the maximum length one step size (user defined) at a time.

#### Primer evaluation and scoring

For each candidate primer, the melting temperature (Tm), GC content (GC), self-complementarity (Self), common SNPs (SNP), tandem repeats (poly), ‘A’s on the 3′ end (EndA), stability of the 3′ end (EnddG) are evaluated. The Tm and self-complementarity’s Tm are calculated using the *oligotm* and *ntthal* programs of the Primer3^6^, respectively; and GC, poly, EndA, and EnddG are counted directly. To check whether the primer sequence contains any common SNPs, a genome file containing common SNPs is created in advance. In this file, the corresponding bases have been changed to “modified degenerate bases” (Supplementary Fig. S1) according to the common SNPs from the dbSNP database^18^ or/and user-defined polymorphic sites. When candidate primers are generated, the corresponding primer sequences containing degenerate bases are also created. The common SNPs of candidate primers can be obtained by parsing the degenerate sequences.

After all these features are evaluated, they are scored separately. One can imagine that a feature should obtain a full score when its value is in the optimal range, and the score gradually decreases to a minimum as the feature value becomes further away from the optimal range. The rate of decrease of the score should be continuous, because a sudden violent decrease in effect of a feature on PCR is impossible in practice. Thus, it is conceivable that the rate of decrease of score changes gradually with the change of the feature value: the rate rises from 0 as the feature value initially moves away from the optimal range, and after the rate reaches the maximum value it gradually goes down to 0, meanwhile, the score reaches the minimum as the feature value goes much farther away. This progress can be modeled by a piecewise logistic function consisting of a high horizontal line in the middle (the optimal range), a logistic curve (a common “S” shaped curve known as the sigmoid curve) on the left, and a symmetric logistic curve on the right (Fig. 2).

The model function in our implementation is defined as follows:

1 $$f(x) = \left\{ {\begin{array}{*{20}l} {\frac{L}{{1 + e^{{ - k(x - x_{0} )}} }} - y_{0} ,} \hfill & {x \le MinO} \hfill \\ {1,} \hfill & {MinO < x \le MaxO} \hfill \\ {\frac{{L^{\prime } }}{{1 + e^{{ - k^{\prime } (x - x_{0}^{\prime } )}} }} - y_{0}^{\prime } ,} \hfill & {x > MaxO} \hfill \\ \end{array} } \right.,$$

where,

$$\begin{array}{*{20}l} {k = \frac{10}{{MinO - MinL}},} \hfill & {k^{\prime} = \frac{10}{{M{\text{ax}}O - MaxL}},} \hfill \\ {x_{0} = \frac{MinO + MinL}{2},} \hfill & {x_{0}^{\prime } = \frac{MaxO + MaxL}{2},} \hfill \\ {y_{0} = \frac{1}{{e^{{ - k(Min - x_{0} )}} }},} \hfill & {y_{0}^{\prime } = \frac{1}{{e^{{ - k^{\prime}(Max - x^{\prime}_{0} )}} }},} \hfill \\ {L = 1 + \frac{1}{{e^{{ - k(Min - x_{0} )}} }},} \hfill & {L^{\prime} = 1 + \frac{1}{{e^{{ - k^{\prime}(Max - x^{\prime}_{0} )}} }},} \hfill \\ \end{array}$$

$L$, $L^{\prime}$ is the maximum value of the curve; $k$, $k^{\prime}$ is the steepness of the curve or the logistic growth rate; $x_{0}$, $x_{0}^{\prime }$ is the *x* value of the sigmoid’s midpoint; $y_{0}$, $y_{0}^{\prime }$ is the *y* offset of the curve; $L$, $k$, $x_{0}$, and $y_{0}$ are constants related to $MinO$, $Min$, and $MinL$; and $L^{\prime}$, $k^{\prime}$, $x_{0}^{\prime }$, and $y_{0}^{\prime }$ are constants related to $MaxO$, $Max$, and $MaxL$. The six feature parameters $MinO$, $MaxO$, $Min$, $Max$, $MinL$, and $MaxL$, denote respectively the minimum and maximum of the optimal range (score equals 1, the full score) of a feature, the minimum and maximum (score equals 0), and the limited minimum and maximum (score reaches the minimum value, usually a negative value).

**Figure 3:**
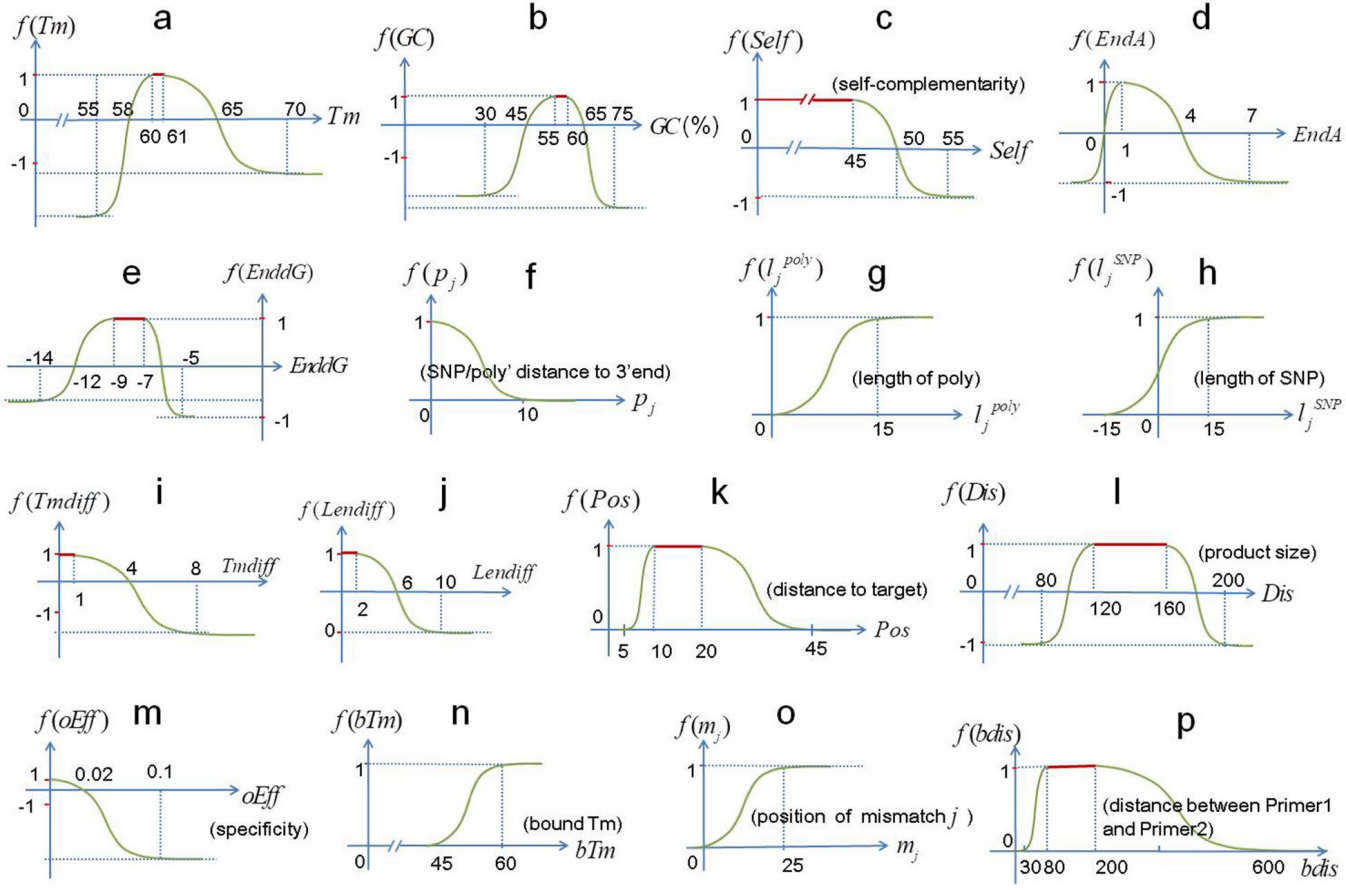
Piecewise logistic model curves of various features. (**a–e**) Score models of Tm, GC, self-complementarity (Self), As on the 3′ end (EndA), and free energy of the 3′ end (EnddG) of primers; (**f–h**) Score models of common SNPs and polys; (**f**) is the model of position (distance to the 3′ end) of the SNP/poly, (**g,h**) are the models of lengths of a poly and SNP, respectively; (**i–m**) Score models of five relation features between primers in a pair: Tm difference, length difference, the distance of the Primer1 to target (Pos), product size, and specificity, respectively. The *x* value of specificity is the sum of efficiencies of all non-target products (oEff). (**n–p**) Efficiency prediction models of a non-target product, n is model of the bound Tm of a primer’s non-target alignment, o is model of position (distance to the 3′ end) of each mismatch in the primer’s alignment, (**p**) is model of the distance between Primer1 and Primer2 in a non-target product.

The function of each feature has its own model parameters: $MinO$, $MaxO$, $Min$, $Max$, $MinL$, and $MaxL$. The default parameters are as follows in order (Fig. 3), which are established from acknowledged experience or literatures and are verified by experiment (Fig. 6f).

- $f(Tm)$: (T, T + 1, T − 2, T + 5, T − 5, T + 10), T is the optimal Tm defined by users;
- $$f(GC)$$: (0.55, 0.6, 0.45, 0.65, 0.3, 0.7);
- $$f(Self)$$: (− 50, 45, − 50, 50, − 50, 55);
- $$f(EndA)$$: (1, 1, 0, 4, − 1, 7);
- $$f(EnddG)$$: (− 9, − 7, − 12, − 6, − 14, − 5).

A primer may contain several SNPs/polys $(\vec{p},\vec{l})$, $\vec{p}$ and $\vec{l}$ are the vectors of positions (distances to the 3′ end of the primer) and lengths of SNPs/polys, respectively. The score of the SNPs/polys is calculated as:

2 $$F(\vec{p},\vec{l}) = 1 - \sum\limits_{j = 1}^{n} {a \cdot \left[ {f(p_{j} ) + b} \right] \cdot f(l_{j} )} ,$$

where $a$ is factor of the score, which equals 4 for SNPs and 2 for polys; $b$ is the base score of an SNP or poly ignoring the effect of its position (distance to the 3′ end), which equals 0.2 for SNPs and 2 for polys; $n$ is the number of SNPs or polys; $f(p_{j} )$ and $f(l_{j} )$ are respectively functions of the position (distance to the 3′ end) and length of SNP/poly $j$. The model parameters are as follows, in order:

- $f(p_{j} )$: (0, 0, 0, 10, 0, 10), applicable to both SNPs and polys.
- $f(l_{j}^{poly} )$: (15,100,0,100,0,100), applicable to polys;where 100 is a large enough length, usually the length of a primer is 18 to 30.
- $f(l_{j}^{SNP} )$: (15,100, − 15,100, − 15,100), applicable to SNPs;where 100 is a large enough length, usually the length of a primer is 18 to 30.

The weighted sum of the scores of all features is calculated as the overall score of a candidate primer. Different weights are set for various features, and the total weight is 100, so the total score of a primer is 100.

#### Pair evaluation and scoring

PrimerScore2 calculates the relation features of all primer pairs with Primer1 and Primer2 in a correct orientation and appropriate distance, including the distance between Primer1 and the target spot (Pos), the distance between Primer1 and Primer2 (Dis, i.e., product size when the type is face-to-face), the difference of length and Tm of Primer1 and Primer2 (Lendiff and Tmdiff), and products (specificity) of the primer pair. The first four features are counted directly, while the specificity is calculated as described below.

Like PrimerBlast^7^, PrimerScore2 aligns primer sequences to databases using BLAST^19^ with a word size of seven and a dynamic, sufficient BLAST expect value cutoff in order to ensure high sensitivity that a target with up to 35% mismatches can be detected. For each alignment, PrimerScore2 uses a modified module from Primer3 *ntthal* to calculate the bound Tm, and then calculates the bound efficiency of the alignment according to Eq. (1). The alignment is filtered if the bound efficiency is lower than a cutoff (default 0.0001).

3 $$E = f(btm) \cdot \prod\limits_{j = 1}^{n} {f(m_{j} )} ,$$

where $n$ is the number of mismatches in the alignment, $f(btm)$ is model of the bound Tm of the alignment, $f(m_{j} )$ is model of distance of mismatch $j$ to the 3′ end. The model parameters are as follows, in order (Fig. 3).

- $f(btm)$: (60, 100, 45, 100, 45, 100). $MaxO$, $Max$, $MaxL$ are large enough, e.g., 100.
- $f(m_{j} )$: (25, 100, 0.95, 100, 0.95, 100). $MaxO$, $Max$, $MaxL$ are large enough, e.g., 100.

When the 3′ end base of the primer is a mismatch, a small amount of product will still be amplified, hence $MinL$ equals 0.95.

**Figure 4:**
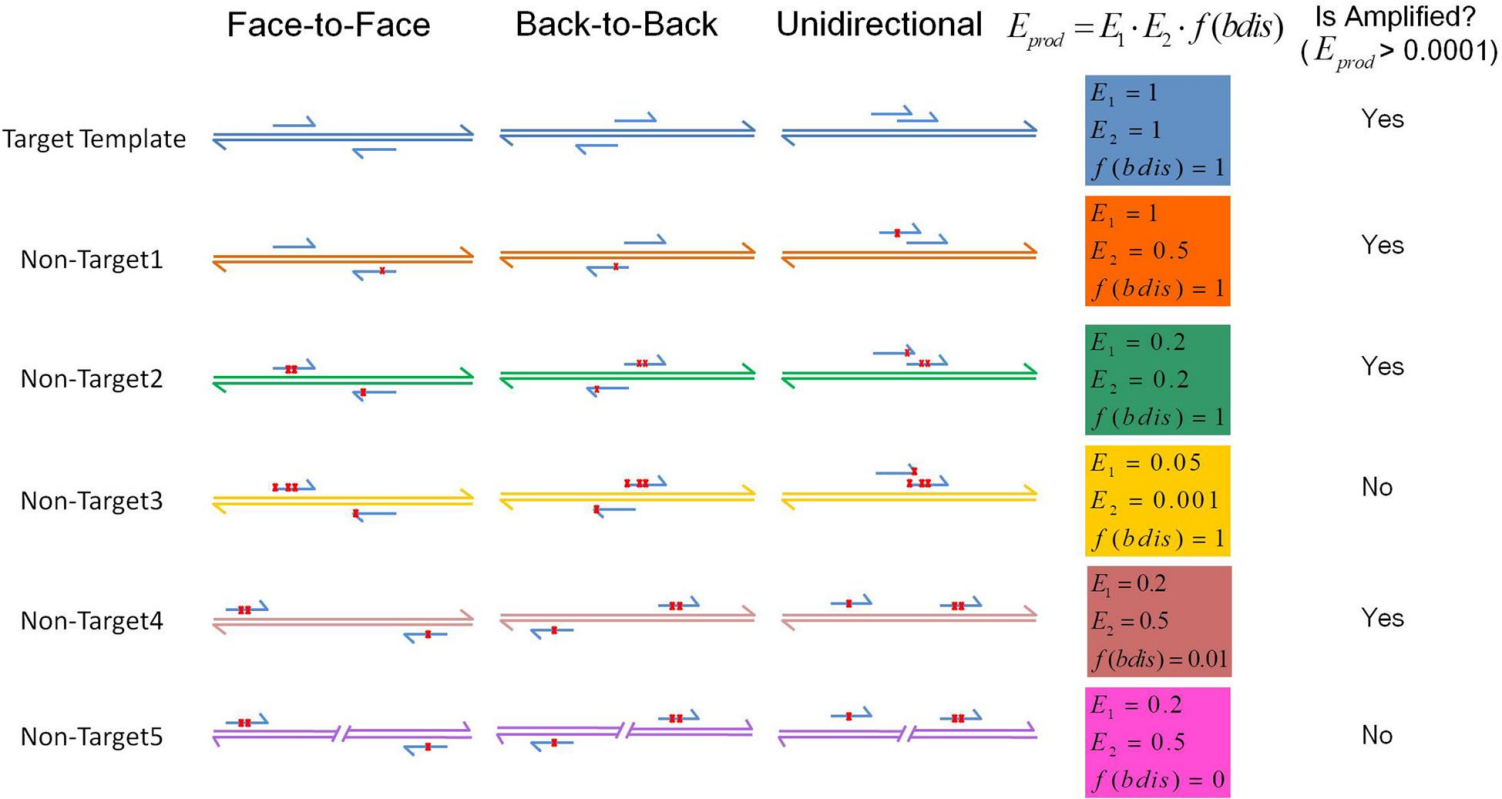
Several examples of the calculation of amplification efficiency of target and non-target products for three kinds of orientation. The factors affecting the amplification efficiency of products are primarily the bound Tm, mismatches (both number and distance to the 3′ end of the primer), and the distance between Primer1 and Primer2 (product size when face-to-face). Two or three examples of each factor were given. The schematic efficiency of each product example ($E_{prod}$) was shown, and the product is amplified only when the efficiency is more than 0.0001.

When Primer1 and Primer2 are aligned on the same template in the correct orientation, and their distance meets the specifications, a product may be amplified. Its amplification efficiency is calculated as follows (Fig. 4):

4 $$E_{prod} = E_{1} \cdot E_{2} \cdot f(bdis),$$

where $E_{1}$ and $E_{2}$ are the bound efficiency of Primer1 and Primer2, respectively, and $f(bdis)$ is model of the distance between Primer1 and Primer2 (product size when face-to-face).

In $f(bdis)$, $MinO$, $MaxO$, $Min$, $Max$, and $MaxL$(maximum PCR size) are defined by the user. The default value of $MinL$ is 30.

If the amplification efficiency is higher than the cutoff (default 0.0001), the product is considered valid. The sum of the efficiencies of all non-target products (oEff), as the value of specificity, is scored using a piecewise logistic model.

After all the relation features have been calculated, they are scored separately using a piecewise logistic model with their own model parameters as follows (Fig. 3), in order:

- $$f(Tmdiff)$$: (0, 1, 0, 4, 0, 8);
- $$f(Lendiff)$$: (0, 2, 0, 6, 0, 10);
- $f(Pos)$: (10, 20, 5, 45, 5, 45) by default, can be defined by the user;
- $f(Dis)$, (120, 160, 100, 180, 80, 200) by default, can be defined by the user;
- $$f(oEff)$$, (0, 0, 0, 0.02, 0, 0.1).

A weighted sum of scores of all relation features is calculated as the score of the relation. Finally, the weighted sum of the scores of Primer1, Primer2, and the relation is the score of the primer pair. The sum of the weights and the full score are all 100.

#### Cross-dimers checking

When designing multiplex primers, cross-dimers among the primers of all targets/templates are checked using Primer3 *ntthal*. By default, three highest-scoring primer pairs of each target/template are checked. Although PrimerScore2 only outputs cross-dimers instead of selecting compatible primer pairs or distributing the pairs to multiple tubes, it remains effective and effortless, because the number of cross-dimers in a 200-plex panel is low enough to be easily avoided by choosing another pair (three pairs have been output) based on years of experimental data.

### Parameters and web-based interface

In order to implement the various design tasks, containing generic primers, genotyping primers, inverse primers, and anchored primers, three important parameters were organized: Primer Type (primer orientation, one of “Face-to-Face,” “Back-to-Back” or “Unidirectional”), Primers Distance (distance between two primers in pair, i.e. product size when primer orientation is face-to-face), and Distance To SNP (distance of first primer to the target spot). By setting these parameters, various tasks can be implemented (Table 1).

**Table 1:** Parameters used to implement different design tasks.

Design tasks^+^	Parameters		
	Primer type	Primers distance**	Distance to SNP**
Generic primer	Face-to-face	120, 160, 80, 200*	–
Sanger genotyping primer	Face-to-face	530, 570, 500, 600*	100, 150, 70, 300*
Arms primer	Face-to-face	120, 160, 80, 200*	0, 0, 0, 0
Inverse primer	Back-to-back	5, 10, 0, 15*	10, 20, 5, 45*
Anchored primer	Unidirectional	− 15, − 10, − 30, − 5*	10, 20, 5, 45*

^+^“Common Primer Type” is named in web-based interface.

*The parameter value is a commonly-used example and it should be set according to the experimental requirement.

**The parameter value consists of four comma-concatenated numbers, which are respectively the minimum and maximum of the optimal range, the minimum and maximum of the permitted range.

**Figure 5:**
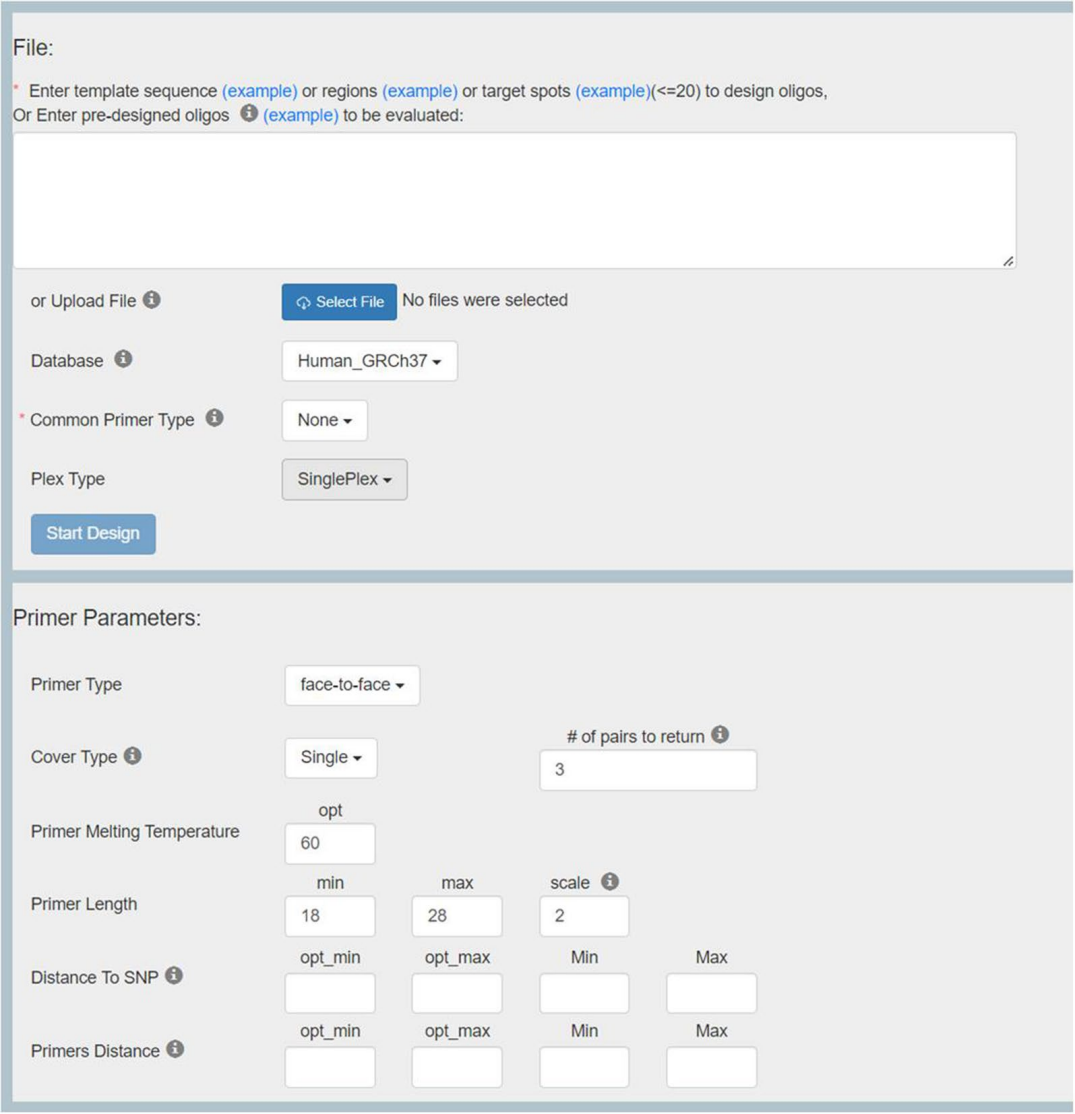
Web-based interface.

PrimerScore2 was written in C++ and Perl. Except a command line version, PrimerScore2 was also developed as an online primer design tool with a simple interface (Fig. 5, http://primerscore.gtxlab.com/). It supports four types of input files: template sequence files in FASTA format, template region files in bed format, target spot files in bed or vcf format, and pre-designed primer list files. An example of all four types will be shown when “example” is clicked. To facilitate the ease of use, “Common Primer Type,” (i.e. design tasks) was developed to simplify the setting of parameters. Selecting one “Common Primer Type,” several important parameters, containing “Primer Type,” “Distance to SNP,” and “Primers Distance,” are set automatically as Table 1 shown, and the parameters added by an asterisk can be changed by users.

## Results

### Design of specificity and score evaluation experiments

The quality of primers mainly depends on two dimensions: specificity and amplification efficiency.

For the specificity, PrimerScore2 aligns the primer template sequences to databases with a sensitivity of up to 35% mismatches, as does PrimerBlast^7^, and then predicts the efficiencies of all possible products of each primer pair. In order to evaluate the accuracy of the efficiency prediction, we selected 12 primer pairs with face-to-face orientation from the human genome with different degrees of specificity—two specific and 10 non-specific, performed gel electrophoresis analysis for each primer pair, and constructed two 12-plex NGS libraries (TM 60 °C, two repetitions) using a standard adaptor ligation protocol library (“Methods” section).

As to the amplification efficiency, PrimerScore2 evaluates and scores various features of primers affecting the amplification efficiency, including Tm, GC, product size, self-complementarity, polys, and 3′ end stability. Assuming high specificity and no common SNPs covered, a primer pair’s score theoretically reflects its amplification efficiency. To evaluate the accuracy of scores predicted by PrimerScore2, 57 primer pairs with different scores were selected to construct two 57-plex NGS experiment (two repetitions), and qPCR for each primer pair were performed.

#### Accuracy of the specificity evaluation

**Figure 6:**
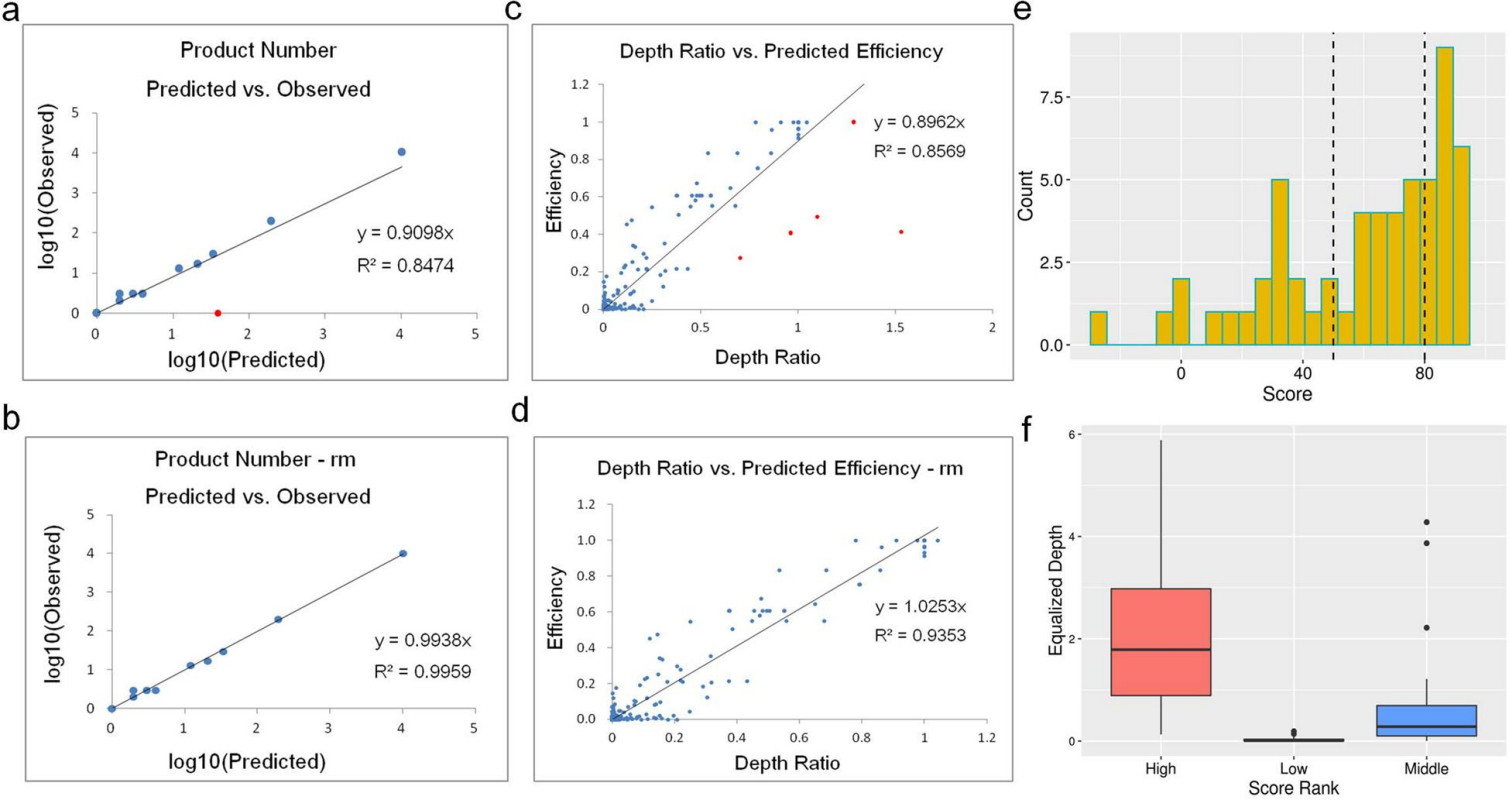
Experimental evaluation of specificity and score in PrimerScore2. (**a,b**) Comparison of observed vs. predicted product number of 12 low-specificity primers. R^2^ (coefficient of determination) reaches 0.99 if an outlier (red point) is removed. (**c,d**) Linear correlation between observed depth ratio and predicted efficiency of all products of 11 low-specificity primers (Speci-6 was excluded for more than 10 k non-target products). The slope of the fitting line was from 0.896 to 1.025, and R^2^ reached 0.94 when five outliers (red points) were removed. (**e**) Distribution of 57 primer scores. Primers with scores of more than 80 were considered to be high-scoring, those with scores lower than 50 were low scoring, and the rest were middle scoring. (**f**) Distribution of the equalized depth of high-scoring, low-scoring, and middle-scoring primers. The equalized depth is the ratio of depth to the mean.

In two 12-plex NGS libraries, we produced average 9 M raw reads in order of high resolution of non-specific primers (Supplementary Table S1). In the NGS data analysis workflow, we filtered them by adapter trimming and primer recognition, then aligned the remained 78.3% reads to genome GRCh38 using BWA^20^, and counted the number of products of all primer pairs and the depths of all target/non-target products, which reflected the specificity of primer pairs and the efficiency of all products, respectively. Here, we showed the result of the first sample, the other repetition had the similar result. Table 2 shows the number of products of 12 primer pairs both observed in NGS and the predicted by PrimerScore2. The depth cutoff of all the non-target products was 50, about 0.001 to 0.0001 of the depth of the target product, and the minimum efficiency cutoff in PrimerScore2 is 0.0001. A primer pair is specific when it has only one product; otherwise it is non-specific. As shown in the table, three primer pairs were observed specific according to the NGS data, while the other nine pairs were non-specific. 11 out of 12 primer pairs’ specificities were predicted accordantly, except Speci-8. We checked the experiment data and found that the depth of Speci-8 is low, 0.065 of the average depth, and it still has 61 minor products with low depths from 1 to 13 (0.000029–0.000377 of its target depth). Figure 6a,b shows a linear correlation between the observed and predicted number of products with an R^2^ value of 0.824, and R^2^ reaching 0.99 when the discrete point Speci-8 was removed.

**Table 2:** Comparison of the number of products observed in NGS and predicted by PrimerScore2 in the 12-plex library.

	Depth	ProductsNum observed	ProductsNum predicted
Speci-1	419,919	1	1
Speci-2	974,725	13	12
Speci-3	58,949	17	21
Speci-4	53,331	2	2
Speci-5	59,762	30	37
Speci-6	2,407,085	10,308	1000+
Speci-7	532,780	3	3
Speci-8	34,459	1	42
Speci-9	1,423,710	201	218
Speci-10	145,668	3	2
Speci-11	194,267	3	3
Speci-12	2,796	1	1

We also counted the depth of each non-target product and its ratio to the depth of the target product, and compared to the predicted efficiency (Supplementary Dataset S1). Figure 6c,d shows a linear correlation between the observed ratios and predicted efficiencies of 11 primer pairs with a slope of 0.896 and R^2^ 0.857. Considering the resolution of the picture and the allowable size of the table, Speci-6 was removed for its too many non-target products. The slope changed to 1.025 and R^2^ reached 0.935 after five outliers were removed. The five outliers were all products of Speci-9 which has the most (200) non-target products and may result from complicated competition and the solution environment: because the number of target/non-target products is relatively large, these products may compete so fiercely for a limited amount of primers that a slight stochastic imbalance of several compositions in solution, such as DNA templates and polymerase, made a difference to the distribution of primers between products and resulted in depth imbalance among all the products.

The gel electrophoresis result (Supplementary Fig. S2) showed that Speci-5 and Speci-6 had diffuse bands and Speci-4 and Speci-9 had an indistinct subband. We examined the fragment sizes of all the products in NGS data, and found that the gel electrophoresis result agreed exactly with the NGS result (Supplementary Table S2), because the most of non-target products had the same fragment size with the target product.

#### Accuracy of the score prediction

In two 57-plex NGS libraries (Supplementary Table S1), average 5 M raw reads were sequenced. After filtering by adapter trimming and primer recognition, about 89.4% reads were remained. Then we counted the depth (reads number) and the equalized depth (divided by the average depth) of all primer pairs, which reflects directly the amplification efficiency. In the 57-plex panel, there were 19 low-scoring pairs with scores less than 50, 19 middle-scoring pairs with scores between 50 and 80, and 19 high-scoring pairs with scores higher than 80 (Fig. 6e). In low-scored pairs, average equalized depth is 0.034, 17 out of 19 (89.5%) pairs’ depth is lower than 0.1, 19 (100%) pairs lower than 0.2. In high-scoring pairs, the average standardized depth was 2.139, and 18 out of 19 (94.7%) pairs had a depth higher than 0.4 (Fig. 6f). Detailed information of the 57-plex experiment is in Supplementary Dataset S1 and Supplementary Fig. S3.

We also performed qPCR for each pair in 57-plex panel, the cycle thresholds (CT) were from 17.7 to 28.0, the average is 22.2, and there was no correlation between CTs and scores, and there was no correlation between CTs and the equalized depths (Supplementary Fig. S4). Therefore, primers with low score are primers of low amplification efficiency and they usually have low depth in unadjusted multiplex panel (high depth could be obtained by increasing the amount of primers), but they can be still effective in monoplex PCR reaction.

### Application to practical projects

It took more than five years to develop and optimize PrimerScore2. During this period, the tool was applied and verified to be effective in more than 10 practical projects: one noninvasive prenatal diagnosis project by cell-free DNA barcode(unique molecule identifier, UMI)-enabled single-molecule test (cfBEST^21^) using multiplex anchored primers, one Sanger sequencing project with monoplex generic primers, one project involving the detection of unknown breakpoints of a large deletion with multiplex fully-covered generic primer pairs, one genotyping project of 159 deafness hotspots with multiplex generic primers, one project to delete virus fusions with unknown partners and more than five projects involving the detection of ultralow frequency tumor mutations and fusions by cfBEST with multiplex anchored primer pairs. The first two projects were described below.

#### Cell-free DNA barcode (unique molecule identifier)-enabled single-molecule test (cfBEST) by multiplex anchored PCR

**Figure 7:**
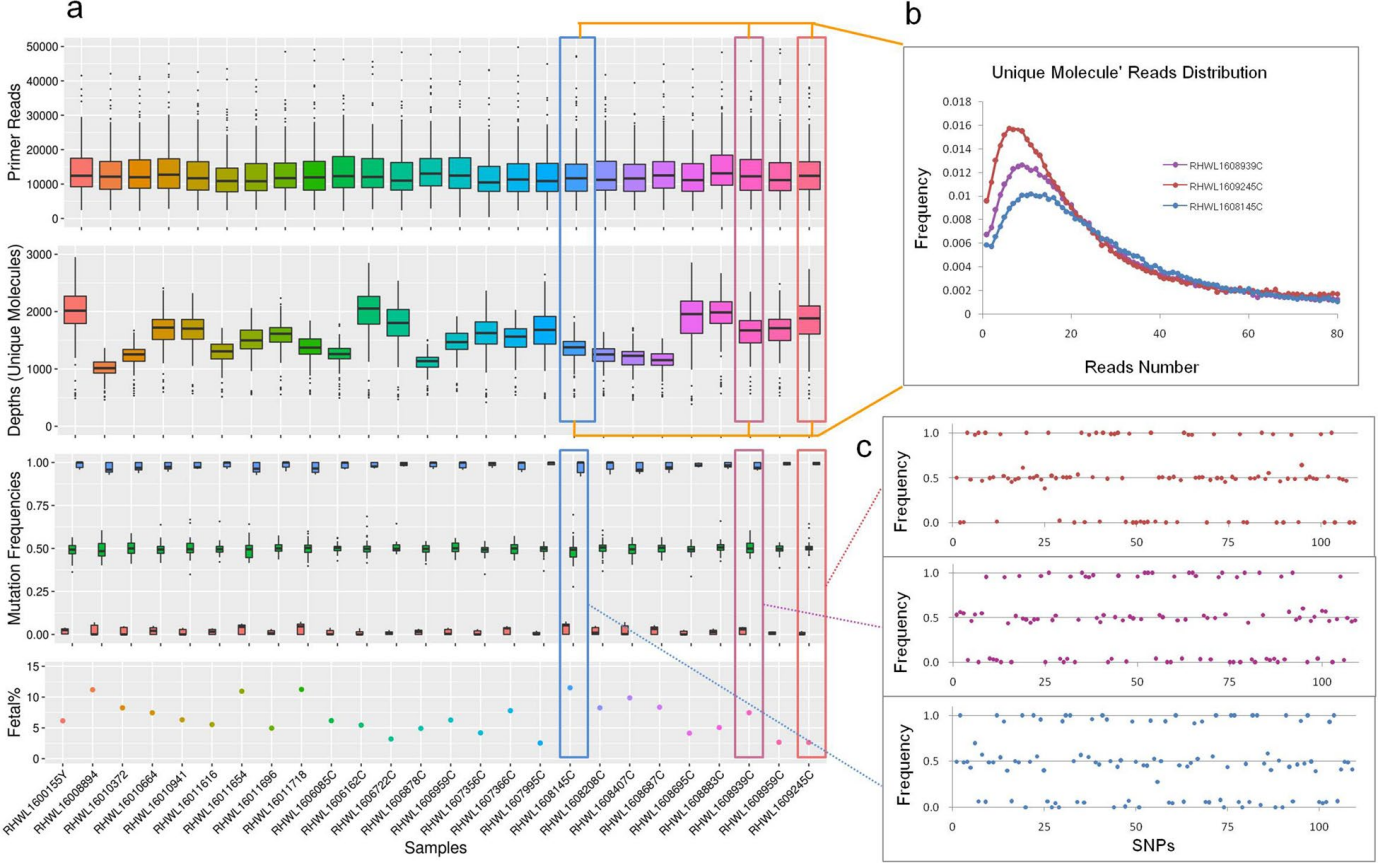
Overall view of 26 samples with male fetuses in cfBEST with multiplex anchored primers. (**a**) Data presentation of 26 samples in four main stages in order of bioinformatic analysis: (1) the distribution of reads number of all SNP primers after the raw data was filtered by barcodes (unique molecules identifiers, UMIs) and primers, (2) distribution of depth (unique molecules number) of all SNPs after calling unique molecules from reads with the same sequences and the same UMIs, (3) distribution of mutation frequencies of all SNPs after calling mutations, (4) presentation of the fetal DNA fractions of all samples. (**b**) Distribution of reads number of unique molecules in RHWL1609245C, RHWL1608939C, and RHWL1608145C with the corresponding colors in figure a. Unique molecules were called from grouped reads with the same sequences and the same unique molecules identity (UMI). (**c**) Presentation of mutation frequencies of 109 SNPs in RHWL1609245C, RHWL1608939C, and RHWL1608145C with the corresponding colors in figure a.

PrimerScore2 was successfully used in a previous study^21^ to design multiplex anchored primers for a cell-free DNA Barcode(unique molecule identifier)-enabled single-molecule test (cfBEST) in noninvasive prenatal diagnosis of β-thalassemia. The goal of the project is to infer fetal genotypes from the mutation frequencies of the β-thalassemia causal sites. Assuming the maternal genotype is heterozygous, theoretically, the frequency is lower than 0.5 when fetal genotype is homozygous negative, more than 0.5 when it is homozygous positive and equals 0.5 when it is heterozygous (Fig. 7c). And fetal DNA fraction is a crucial factor which affects the frequency offset amplitude. To calculate the fetal DNA fraction, we selected 116 SNPs with high heterozygosity (minor allele frequency, MAF close to 0.5) in the Chinese population. PrimerScore2 was used to design 232 multiplex anchored primer pairs, one pair on the upstream and one pair on the downstream of each SNP. After two preliminary experiments, 7 SNPs are filtered on account of low or too high depth. Finally, coupled with 13 common β-thalassemia causal sites, the remained 109 SNPs were captured in two tubes respectively for 116-plex upstream and 114-plex downstream anchored PCR panel after the original DNA templates was enabled with barcodes, i.e. unique molecules identifiers (UMI).

**Figure 8:**
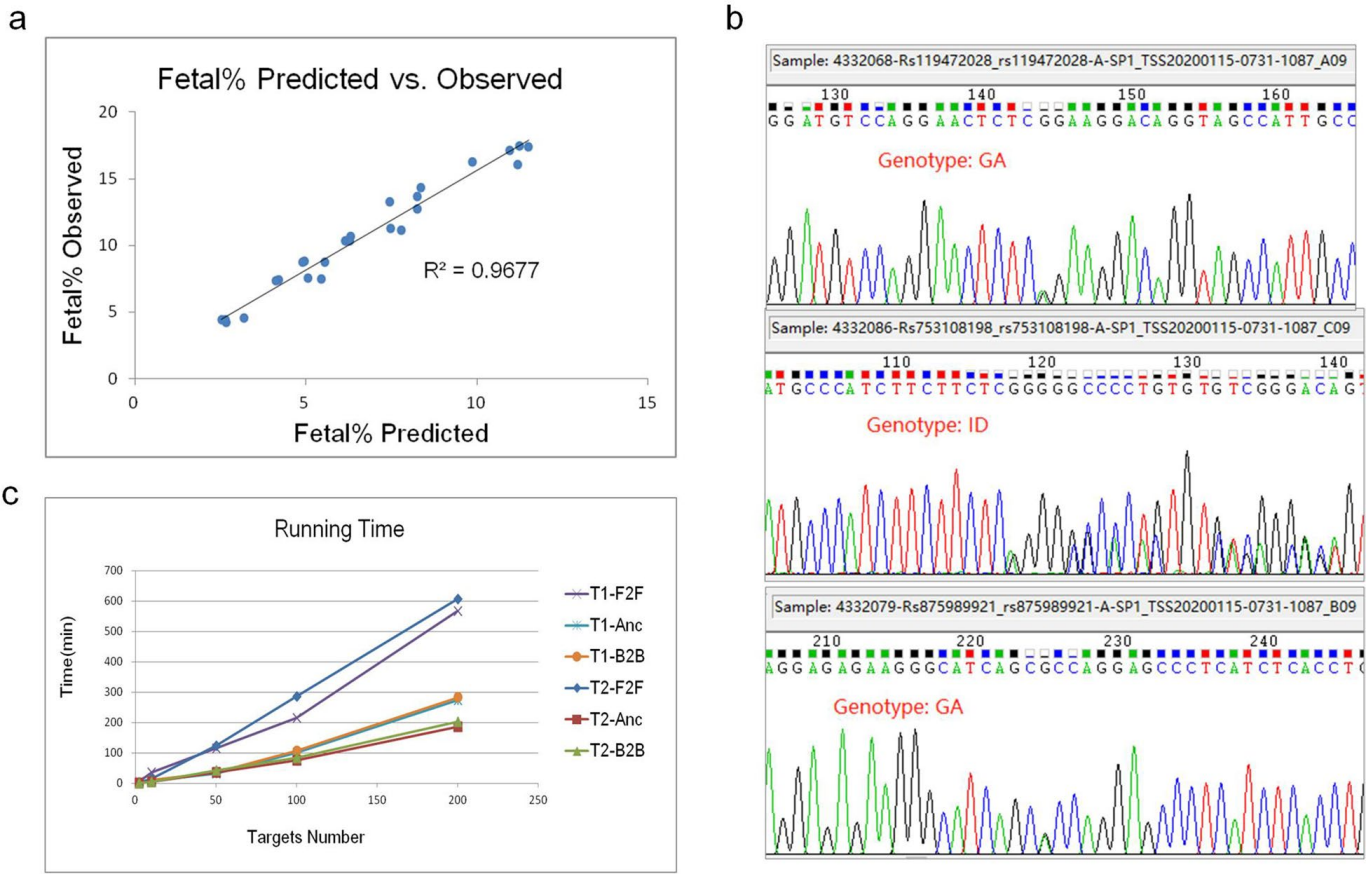
Evaluation of accuracy of the predicted fetal DNA fraction, Sanger sequencing results, and evaluation of running time of PrimerScore2. (**a**) Comparison of predicted fetal DNA fraction in cfBEST and observed by the gold standard method (Y-assay). (**b**) Sanger sequencing results of the three examples in Sanger sequencing genotyping project. The genotypes were GA, ID (‘I’ is insertion; ‘D’ is deletion) and GA, respectively. (**c**) Running time of three primer orientations (*F2F* Face-to-Face, generic primers, *Anc* Anchored primers, *B2B* Back-to-Back, inverse primers) in five ranks of target numbers: 2, 10, 50, 100, and 200. Two target sets (T1 and T2) were tested.

We compared cfBEST with the gold standard method for fetal DNA fraction determination—the relative proportion of mapped Y chromosomal fragments (Y-assay). We applied cfBEST to 26 maternal plasma samples from pregnancies with karyotyping-confirmed male fetuses, in which cell-free fetal DNA proportion had been measured according to Y-assay (Supplementary Tables S3, S4). Average 12 M reads for each sample were sequenced, about 88.7% were remained after filtering by barcodes (UMIs) and primers, reads number of the SNPs’ primers were average 13 k (Fig. 7a) and of the thalassemia sites’ primers were 357 k (the higher reads number of thalassemia sites’ primers was because the amount of the primers was increased to improve the accuracy of genotyping of the thalassemia sites). Finally, after calling unique molecules from grouped reads with the same sequences and the same UMI, whose depth (number of grouped reads) peaks were around 10 (Fig. 7b), average 1507 unique molecules for SNPs and average 2510 unique molecules for thalassemia sites were used to calculate mutation frequencies and to infer the fetal DNA fraction (Fig. 7a,c, Supplementary Dataset S2). The calculated fractions were highly concordant with those from the Y-assay with a coefficient of determination (R^2^) 0.97 (Fig. 8a).

In the previous study, cfBEST was applied to a blind validation study consisting of 143 β-thalassemia at-risk pregnancies, high sensitivity and specificity proved the validity of cfBEST, and also the validity of both the 116-plex and 114-plex anchored primers by PrimerScore2.

#### Sanger sequencing project by monoplex generic primers

PrimerScore2 was used to design 77 monoplex Sanger sequencing primer pairs targeting 80 SNPs across 91 samples with face-to-face orientation, and a total of 151 Sanger sequencing events were performed. The Sanger sequencing results (Fig. 8b) indicated that all primers were effective and all SNPs were successfully amplified and sequenced. The target, primer, and genotype files are shown in Supplementary Dataset S3.

### Running time

In order to evaluate the specificity of primers, PrimerScore2 is required to align the template sequences to database using BLAST^19^, and then is needed to infer all target/non-target products for a mass of candidate primer pairs. This process is expensive in terms of time. PrimerScore2 performs well after years of development. We assessed using 10 threads in a 280G RAM computer targeting 2, 10, 50, 100, and 200 human SNPs for generic, inverse, and anchored primers. The running time of inverse and anchored primers was about 0.5–1 min per target (SNP, indel), and the running time of generic primers was about twice that (Fig. 8c).

### Comparison with other primer design tools

PrimerScore2 has several advantages over other primer design tools (Table 3).

**Table 3:** Comparison of PrimerScore2 and other design tools.

	PrimerScore2	Ultiplex	PrimerPlex	Oli2go	MPD	Primer-Mapper	MFEprimer-3.0	MPprimer	Batch-Primer3	Primer-BLAST	Primer3
Input|designing on templates	Fasta, Bed	Bed	Bed, Gene/exon	Fasta	Bed	Fasta, GenBank	Bed	Fasta	Fasta	Fasta, RefSeq	Fasta
Input|designing for targets	Bed, Vcf	Bed	CDS list	×	×	Fasta1, dbSNP	Bed	Fasta2	Fasta1	×	Fasta2
Input|evaluating pre-designed primers	Primers list	×	×	×	×	×	Primer sequences	×	×	Primer sequences	Primer sequences
Selection strategy	Score	Filtration	Filtration	Filtration	Filtration	Filtration	Filtration	Primer3	Score2	Primer3	Score1
Supportive plex type	Mono/Multiplex	Mono/Multiplex	Mono/Multiplex	Multiplex	Mono/Multiplex	Mono/Multiplex	Mono/Multiplex	Mono/Multiplex	Mono/Multiplex	Monoplex	Monoplex
Specificity evaluation	√	√	√	√	√’	√	√	√	×	√	×
Common SNPs check	√	√	√	×	√	×	√	×	×	×	×
Crossdimer check	√	√	√	√	√	×	√	√	×	×	×
Web interface	√	√	×	√*	√*	√*	√	√*	√*	√	√

Fasta1: Variant FASTA with a degenerate SNP in the middle of the sequence.

Fasta2: FASTA, targets are specified by position on the sequence and length.

Score1: Calculating penalty score only for basic features, not for specificity or common SNPs.

Score2: Calculating score only for basic features, not for specificity or common SNPs.

√’: By quality score of *k*-mer uniqueness.

√*: Connection failed.

It can design both monoplex and multiplex primer pairs that are aimed at both template sequences (pairs on the templates) and targets (pairs flanking the targets). It is the only tool that can design inverse primer pairs with a back-to-back orientation, and the only tool that can design pairs that cover the whole target region evenly. It is also one of the few fully automatic tools that can finish the design task in one click.

Most primer design tools check and filter candidate primers feature by feature, as a result, design failures frequently occur and it is necessary to repeatedly loosen parameters and start the design again and again, this brings a great of trouble and time consumption to researchers. PrimerScore2 uses a scoring method to select primers and returns the highest-scored primers with no failure, and it is the only one to score all features containing specificity and common SNPs. PrimerScore2 is the only tool that can predict the amplification efficiency of each non-target product, and one of the few tools providing an accessible web interface.

## Discussion

We present PrimerScore2, a robust primer design tool for mono- and multiplex panels. It can design multiple types of primers for several PCR variants, it selects primers based on their comprehensive scores, a weighted sum of all features’ scores inferred using a piecewise logistic model, so that the issues of design failure and the need to relax the parameters to re-start design, which occur in most design tools, is avoided. It offers a one-click solution to the design task, and saves researchers time.

It took more than 5 years to develop and optimize PrimerScore, and a big upgrade was carried out two years ago so that PrimerScore2 is named now. This long-term optimization and abundant application made PrimerScore2 a robust and precise tool applicable to multiple PCR variants. It has been verified to be effective and robust by more than 10 practical projects, including one monoplex Sanger sequencing project, one genotyping project of deafness hotspots by multiplex generic primer pairs, one project aimed at the detection of unknown deletion positions by generic fully-covered primer pairs, one project on fetal genotyping of thalassemia hotspots^21^ by multiplex anchored primer pairs, one project to delete virus fusions with unknown partners by multiplex anchored primer pairs, and more than five projects to detect ultralow frequency tumor mutations by multiplex anchored primer pairs. The plex numbers in the multiplex projects were mostly 50-plex to 200-plex. Although PrimerScore2 only outputs cross-dimers, instead of selecting compatible primer pairs or automatically distributing the pairs to multiple tubes, it remains effective and easy to use because the number of cross-dimers within a 200-plex is low enough to be easily avoided by choosing other pairs from the three pairs output, based on our experience in the above projects. Note that the amount of dimer produced in experiment is related to the polymerase used and primer’s phosphorothioate modification, the phosphorothioate primers and Phusion® HF DNA Polymerase (NEB, USA) were used in all of the above projects. In the more than 10 projects, only the genotyping project of deafness hotspots encountered serious cross dimers, the primers were finally distributed to two tubes. This may be because deafness hotspots are dense and the template regions are complicated. Primer3-*ntthal* could successfully detect the major dimers in our projects, but it seems that the sensitivity and specificity of the minor dimers could not be balanced well. Dimer bands in electrophoretograms and dimers with moderate and low depth in NGS analysis usually cannot be detected, loosening various parameters could increase the sensitivity, but led to the identification of many false dimers. The optimization of dimer detection is one of our priorities for the future.

PrimerScore2 is intended to become an effective and fully-automated primer design tool suitable for a variety of PCR applications, which frees researchers from tedious tool selection and manual design. PrimerScore2 is already capable of designing methylation-specific primers and probes, and several new functions may be added in the future, including bisulfite sequencing PCR pairs, primer compatibility determination and distribution in multiplex panels, and possibly transcript primers for expression quantification and degenerative primers for virus and bacterial detection.

12-plex and 57-plex NGS experiments dedicated to the optimization and verification of PrimerScore2 made a great contribution. PrimerScore2 previously checked specificity using BWA^20^ to align candidate primers to databases and calculated the bound TM of non-target regions using Primer3-*ntthal*. During analysis of a 12-plex library, we found that many alignments of primers containing highly repetitive sequences are missed by BWA, and Primer3-*ntthal* contains a bug that an error in thermodynamic parameter leads to errors in alignment and a false bound Tm. Hence, PrimerScore2 has been optimized to use BLAST to align template sequence to databases and pick the source code of Tm calculations from Primer3-*ntthal* to calculate bound Tm. We also found that a mismatch at the 3′ end base will not completely prevent PCR amplification, and a few products will still be created. PrimerScore2 has been upgraded in the light of this discovery. The 57-plex NGS experiment made a great contribution to the parameters of piecewise logistic models and weights of various features. And its analysis indicated that some features, such as Tm and GC, have a major effect on the amplification efficiency of primer pairs, while some have little effect, including the number of ‘A’s at the 3′ end of primers, and the Tm and length difference between two primers in one pair. A suitable product size, high Tm, and poly G on the 3′ end of primers will increase the amplification efficiency.

## Methods

### Sample acquisition and gDNA extraction

Blood samples were collected with consent from subjects at the Nanfang Hospital, Southern Medical University. DNA extraction from 200 μL of whole blood samples was conducted in accordance with the instructions of the Whole Blood Genome DNA Extraction Kit (Changzhou, China) using the bead purification method (Genmagbio, Beijing).

### Primer amplification and library construction

The primer concentration was 10 μM in a ratio of 1:1 of primer pairs. The first round of total PCR reaction volume was 50 μL:1.5 μL of Phusion^®^ HF DNA Polymerase (NEB, USA), 10 μL of 5 × Phusion^®^ GC Reaction Buffer, 4 μL of dNTP Mixture (2.5 mm each), 3 µL of 100% DMSO, 2 µL of MgCl2 (50 mM), 20 ng of gDNA template, 2 µL of primer mix, to a final concentration of each pair of primers in the system of 0.7 nM, and distilled deionized water. The first round of the PCR reaction was performed at 95 °C for 10 min, followed by 30 cycles of 95 °C for 30 s, 60 °C for 30 s, 72 °C for 30 s, and then a final step at 72 °C for 7 min. The PCR products were then purified with AMPure XP beads (Agencourt, MA, USA), and 21 µL of the purified product was taken as the template for the second round of the PCR reaction. The PCR reaction volume included 25 µL of HiFi HotStart ReadyMix (Roche Diagnostics, Basel, Switzerland), 0.5 µL of universal connector primers i5 and i7 (25 µM), and 3 µL of 100% DMSO. The amplification procedure was started at 98 °C for 3 min, followed by 3 cycles of 98 °C for 10 s, 52 °C for 30 s and 8 cycles of 98 °C for 10 s, then 72 °C for 1 min. Finally, 72 °C for 5 min. The PCR product was purified using AMPure XP magnetic beads (Agencourt) and the nucleic acid concentration was determined using a Qubit 2.0 (ThermoFisher Scientific, Waltham, MA, USA) and 2% agarose electrophoresis gel. Qualified libraries were sequenced on Illumina’s NovaSeq platform.

### Sequencing and analysis

The 12-plex and 57-plex libraries were sequenced using the Illumina NS500 and NovaSeq 6000 Systems, respectively. After low-quality and adapter filtering using Flexbar v2.5, the clean reads were used to recognize the primers depending on which primer the start (5′ end) sequence on the read was the same as, and then were mapped to the human genome (GRCh38) using BWA 0.7.11. Finally, we counted the number of products of each primer pair and the depth of each product for the 12-plex library. For the 57-plex library, as the number of products of 57 primer pairs is almost always 1, we only calculated the total depths of the primer pairs.

### Ethics declarations

All procedures performed in studies were approved by the Internal Ethics Committee of Southern Medical University. All methods were carried out in accordance with relevant guidelines and regulations. Informed consent was obtained from all participants.

## Supplementary Information

Supplementary Information 1.

Supplementary Information 2.

Supplementary Information 3.

Supplementary Information 4.

## Data Availability

The raw NGS data analysed during the current study are available in the EMBL-EBI repository: PRJEB55712. The primer sequences for all experiments can be found in Supplementary Dataset.

## Code availability

The source code can be downloaded from https://github.com/Genetalks/PrimerScore2, the web-based interface is on http://primerscore.gtxlab.com/.
