# Supplementary material for: High-throughput primer design by scoring in piecewise logistic model for multiple polymerase chain reaction variants
Source: Sci Rep. 2022 Dec 7;12:21136. doi: 10.1038/s41598-022-25561-z (PMC9729204; doi:10.1038/s41598-022-25561-z)

**Supplementary Table S1. Primers statistical results of 4 NGS libraries for specificity and score evaluation.** “UnkownPrimer” were reads with unkown primers, i.e. the reads didn’t contain any primers; “NoPair” were reads with primers in different pairs, e.g. one read contained primer A and the other read contained primer B; “PrimerPair” were reads with the right primers in one pair, which were used in the subsequent analysis.

| **Library** | **Panel** | **RawReads** | **UnkownPrimer** | **UnkownPrimerRatio** | **NoPair** | **NoPairRatio** | **PrimerPair** | **PrimerPairRatio** |
| --- | --- | --- | --- | --- | --- | --- | --- | --- |
| SDDF2021101203 | 12-plex | 8,773,746 | 1,116,557 | 12.73% | 805,369 | 9.18% | 6,851,820 | 78.09% |
| SDDF2021101204 | 12-plex | 9,599,436 | 1,211,684 | 12.62% | 851,925 | 8.87% | 7,535,827 | 78.50% |
| SDDF2021101210 | 57-plex | 9,119,163 | 786,957 | 8.63% | 250,372 | 2.75% | 8,081,834 | 88.62% |
| SDDF2021101216 | 57-plex | 8,961,839 | 716,665 | 8.00% | 161,295 | 1.80% | 8,083,879 | 90.20% |

**Supplementary Table S2. Summaries of gel and NGS results of 12 primers for specificity evaluation and comparison to the predicted specificity.** Full-length gel figure saw supplementary figure S2.

| **PrimerID** | **Gel (TM: 55°C, 60°C, 65°C)** | **NGS** | **Gel vs. NGS** | **Predicted vs. Gel** |
| --- | --- | --- | --- | --- |
| Speci-1 | Only one target band in all TMs. | One product with depth 15593X. | Accordant | Accordant |
| Speci-2 | Only one target band in all TMs. | Several products, target product size is 160bp and non-target product sizes are about 130bp with low depth. | Accordant ne-arly | Accordant |
| Speci-3 | Only one target band in all TMs. | Several products with size all about 150bp. | Accordant | Accordant |
| Speci-4 | A subband in TMs lower than 60°C. | Two products, the target product size is 182bp, the other product size is 94bp. | Accordant | Accordant |
| Speci-5 | Severe dispersion, about five subbands above the target band and one subband down can be distinguishable. | Many products, target product size is 150bp, others are 110bp, 230bp, 400bp…. | Accordant | Accordant |
| Speci-6 | Light dispersion, no subband can be distinguishable. | A great many of products, their sizes are mainly about 130bp, some 120bp, 150bp, 160bp, 270bp…. | Accordant | Accordant |
| Speci-7 | Only one target band in all TMs. | Three products with the same fragment size of 141bp. | Accordant | Accordant |
| Speci-8 | Only one target band in all TMs. | Only one product. | Accordant | Accordant nearly |
| Speci-9 | A subtile subband above the target band. | Several products with main sizes about 110bp and one 397bp. | Accordant | Accordant |
| Speci-10 | Only one target band in all TMs. | Mainly one product, other products' depths were extremely low. | Accordant | Accordant |
| Speci-11 | Only one target band in all TMs. | Mainly one product, other products' depths were extremely low. | Accordant | Accordant |
| Speci-12 | Only one target band in all TMs. | Only one product with low depth 2000X. | Accordant | Accordant |

**Supplementary Table S3. Bioinformation statistical results of** **26 maternal plasma samples with male fetuses in cfBEST.** “LowDepth” were reads in unique molecules of depth lower than cut-off; “ConsensError” were reads in unique molecules with too many errors in sequences; “DupRatio” were duplicate proportion; “AverDepth” were average unique molecules number on all target sites (109 SNPs and 13 β-thalassemia sites).

| **Sample** | **RawReads** | **UMIErrorRatio** | **PrimerRatio** | **InsertSize** | **LowDepth** | **ConsensError** | **DupRatio** | **AverDepth** |
| --- | --- | --- | --- | --- | --- | --- | --- | --- |
| RHWL1606959C | 13807680 | 5.41% | 89.67% | 131.19 | 1.64% | 1.26% | 98.06% | 1549 |
| RHWL1608695C | 12429411 | 5.85% | 89.67% | 135.32 | 3.56% | 1.22% | 97.03% | 2134 |
| RHWL1608208C | 13085977 | 5.94% | 89.90% | 138.33 | 1.36% | 1.18% | 98.26% | 1293 |
| RHWL16008894 | 13888990 | 5.64% | 89.93% | 135.35 | 1.09% | 1.10% | 98.65% | 1061 |
| RHWL1607995C | 12093163 | 6.12% | 89.82% | 136.6 | 3.60% | 1.24% | 97.30% | 1915 |
| RHWL1607356C | 11992817 | 6.01% | 88.97% | 135.08 | 2.48% | 1.01% | 97.43% | 1760 |
| RHWL1600155Y | 13500465 | 6.46% | 89.46% | 135.23 | 3.18% | 1.35% | 97.10% | 2239 |
| RHWL16011654 | 12774133 | 5.79% | 89.26% | 131.58 | 2.26% | 1.02% | 97.77% | 1590 |
| RHWL1606722C | 12028881 | 6.39% | 89.39% | 139.37 | 2.85% | 1.34% | 97.18% | 1937 |
| RHWL1606162C | 13840978 | 5.93% | 89.43% | 132.56 | 3.08% | 1.33% | 97.21% | 2209 |
| RHWL1606085C | 14056352 | 5.52% | 89.56% | 138.56 | 1.30% | 1.22% | 98.35% | 1315 |
| RHWL1608883C | 14386317 | 5.99% | 89.82% | 131.18 | 2.16% | 1.39% | 97.48% | 2084 |
| RHWL16010941 | 13390633 | 6.14% | 89.68% | 134.73 | 2.17% | 1.25% | 97.65% | 1791 |
| RHWL16010372 | 14306636 | 6.16% | 89.27% | 125.8 | 1.29% | 1.08% | 98.39% | 1296 |
| RHWL1609245C | 11181083 | 7.35% | 87.77% | 121.94 | 3.38% | 1.17% | 96.65% | 1666 |
| RHWL1608939C | 11328689 | 6.38% | 87.77% | 125.02 | 2.28% | 1.03% | 97.18% | 1420 |
| RHWL1607366C | 10772614 | 7.30% | 87.88% | 122.63 | 2.27% | 1.23% | 97.24% | 1303 |
| RHWL16011696 | 9909256 | 6.27% | 87.10% | 136.18 | 2.43% | 1.17% | 96.93% | 1347 |
| RHWL16011616 | 9817593 | 7.77% | 87.08% | 143.85 | 2.04% | 1.16% | 97.48% | 1090 |
| RHWL1606878C | 11270858 | 7.44% | 87.16% | 137.68 | 1.35% | 1.18% | 98.09% | 942 |
| RHWL1608687C | 10928586 | 6.60% | 87.53% | 136.66 | 1.35% | 1.04% | 98.00% | 963 |
| RHWL16010664 | 11491987 | 7.02% | 87.89% | 120.2 | 2.43% | 1.17% | 97.12% | 1461 |
| RHWL1608145C | 10799720 | 6.43% | 88.15% | 119.87 | 1.82% | 0.97% | 97.59% | 1147 |
| RHWL16011718 | 11125746 | 7.18% | 87.98% | 124.58 | 1.68% | 1.04% | 97.64% | 1146 |
| RHWL1608407C | 9979020 | 7.00% | 87.77% | 129.95 | 2.61% | 1.18% | 97.59% | 1052 |
| RHWL1608959C | 10754718 | 7.77% | 88.12% | 126.31 | 2.95% | 1.22% | 96.90% | 1468 |

**Supplementary Table S4. Sample information of 26 maternal plasma samples with male fetuses in cfBEST.**

| **Sample** | **Maternal Age**  **(year)** | **Gestational Age**  **(week)** | **Fetal%** |
| --- | --- | --- | --- |
| RHWL1600155Y | 39 | 17 | 10.33 |
| RHWL16008894 | 26 | 18 | 16.02 |
| RHWL16010372 | 31 | 18 | 13.63 |
| RHWL16010664 | 34 | 19 | 13.26 |
| RHWL16010941 | 36 | 17 | 10.66 |
| RHWL16011616 | 40 | 13 | 8.71 |
| RHWL16011654 | 25 | 18 | 17.14 |
| RHWL16011696 | 20 | 16 | 8.79 |
| RHWL16011718 | 31 | 19 | 17.44 |
| RHWL1606085C | 36 | 16 | 10.36 |
| RHWL1606162C | 41 | 13 | 7.49 |
| RHWL1606722C | 35 | 14 | 4.51 |
| RHWL1606878C | 41 | 20 | 8.73 |
| RHWL1606959C | 26 | 13 | 10.35 |
| RHWL1607356C | 28 | 16 | 7.39 |
| RHWL1607366C | 23 | 15 | 11.13 |
| RHWL1607995C | 26 | 17 | 4.38 |
| RHWL1608145C | 37 | 20 | 17.41 |
| RHWL1608208C | 39 | 12 | 12.73 |
| RHWL1608407C | 37 | 25 | 16.27 |
| RHWL1608687C | 35 | 15 | 14.31 |
| RHWL1608695C | 30 | 15 | 7.34 |
| RHWL1608883C | 27 | 22 | 7.56 |
| RHWL1608939C | 35 | 18 | 11.23 |
| RHWL1608959C | 35 | 20 | 4.2 |
| RHWL1609245C | 32 | 17 | 4.41 |

**Supplementary Figure S1.** **Schematic conversions to “****modified degenerate bases” when the bases in the genome contained common SNPs, short inserts or deletions.** The bases with SNPs were changed to acknowledged degenerate bases, the bases deleted were replaced to ‘E’, and the bases with inserts with length of 1bp, 2bp, and more than 3bp were changed to ‘I’, ‘J’ or ‘L’, respectively.


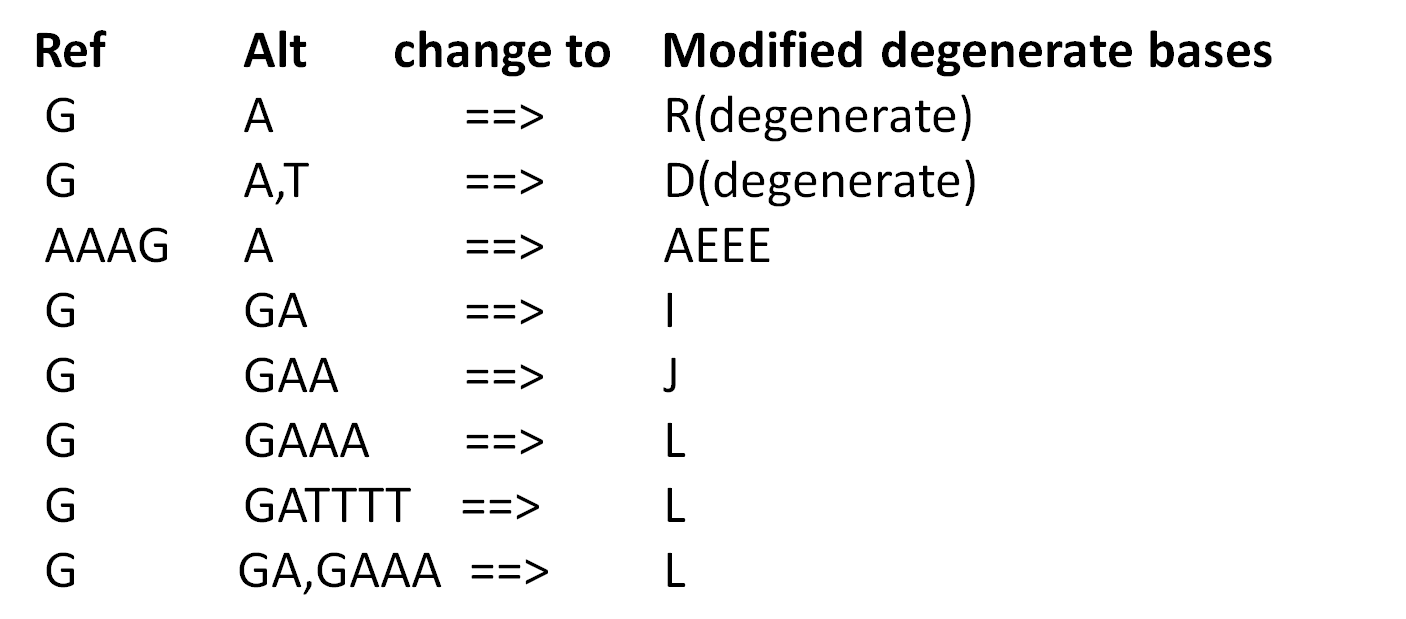


**Supplementary Figure S2. The original electrophoretic gel of 12 primers used for specificity evaluation in TM 55°C, 60°C, and 65°C.** The 12 bands between the first marker (the first band on the top) and the second marker (the 14th band on the top) were evaluated in 55**°**C, the 12 bands between the second marker and the third marker (the second band on the bottom) were evaluated in 60**°**C, and followed by 12 bands evaluated in 65**°**C; all the bands were of 12 primers in number order. The bands in bottom right corner were not relevant to this study.


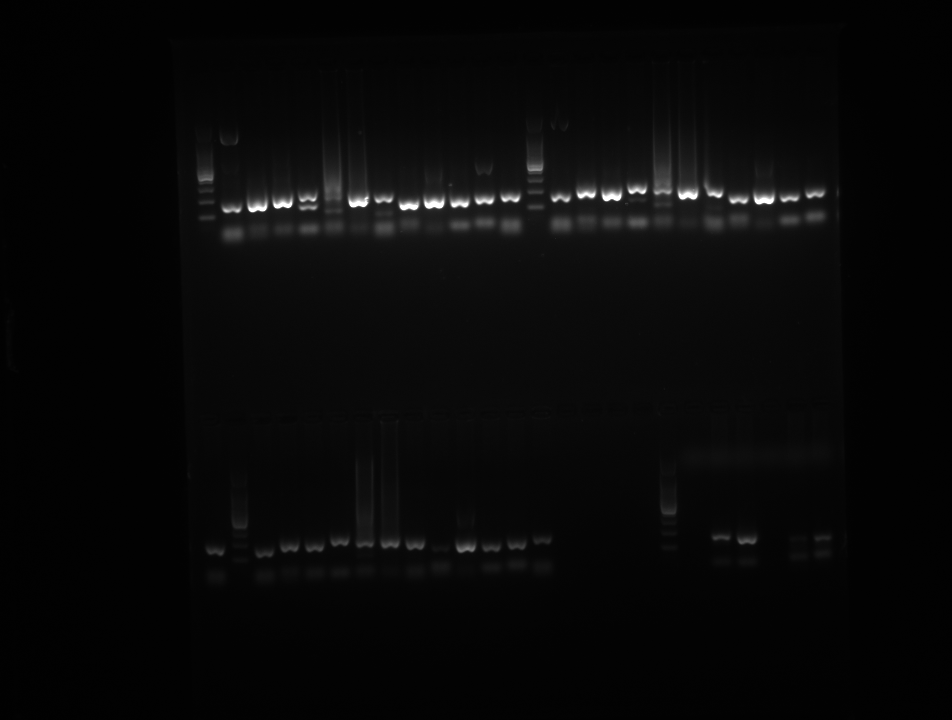


**Supplementary Figure S3**. **Heatmap of score, depth and various normalized features of 57 primers.** Primers are sorted by score in descending order. “1” and “2” after feature names indicated the forward and reverse primer, respectively.


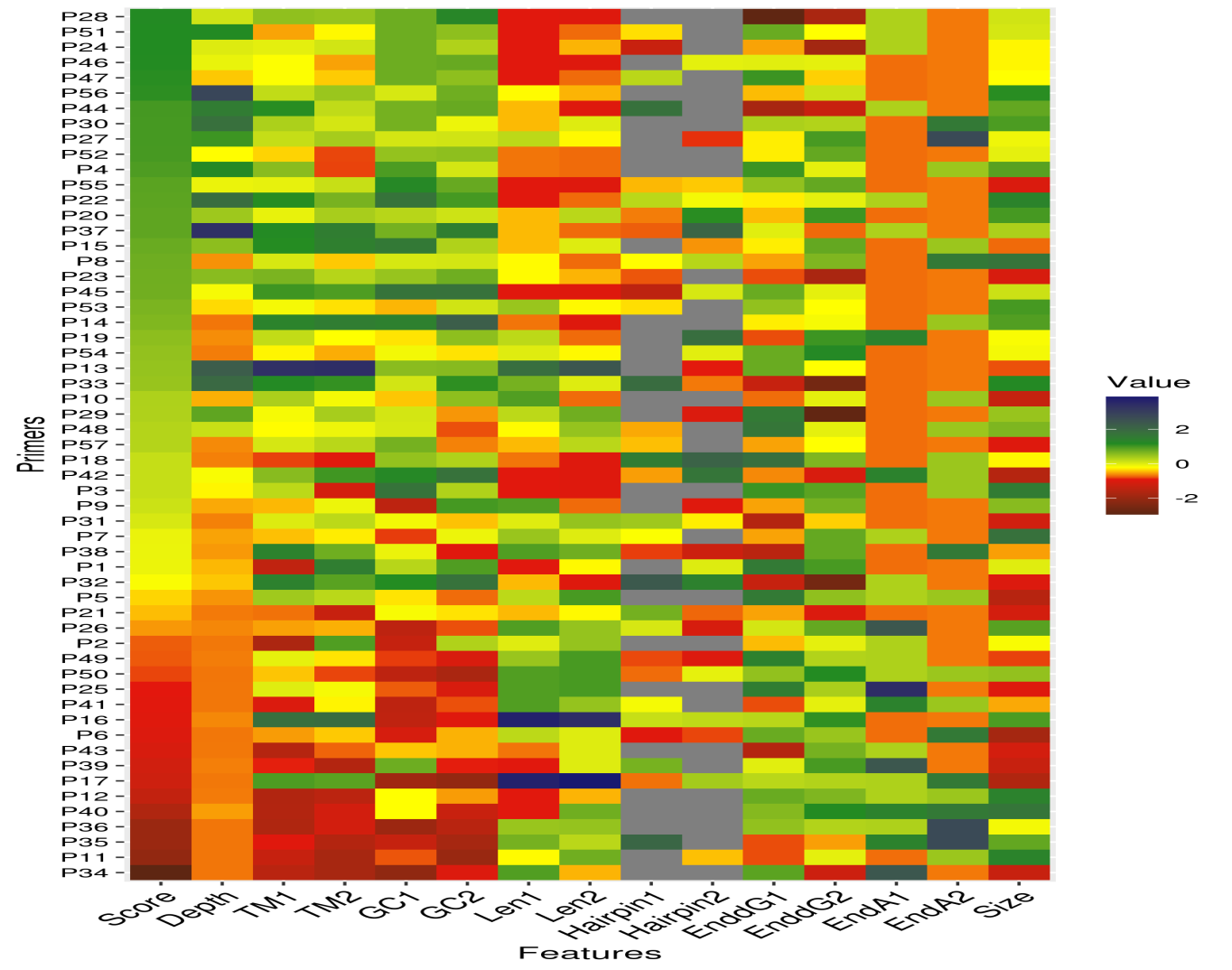


**Supplementary Figure S4. Cycle thresholds (CTs) and exampled amplification plots of qPCRs of 57 primers for score evaluation. a** Comparison of CTs and equalized depth of 57 primers. There is no correlation between CTs and equalized depths. **b** Comparison of CTs and scores of 57 primers. There is no correlation between CTs and scores. **c** Amplification plots of P34 (purple, score=-28.1) and P35 (pink, score=0.4). The primers with low scores can be effective in monoplex reaction.


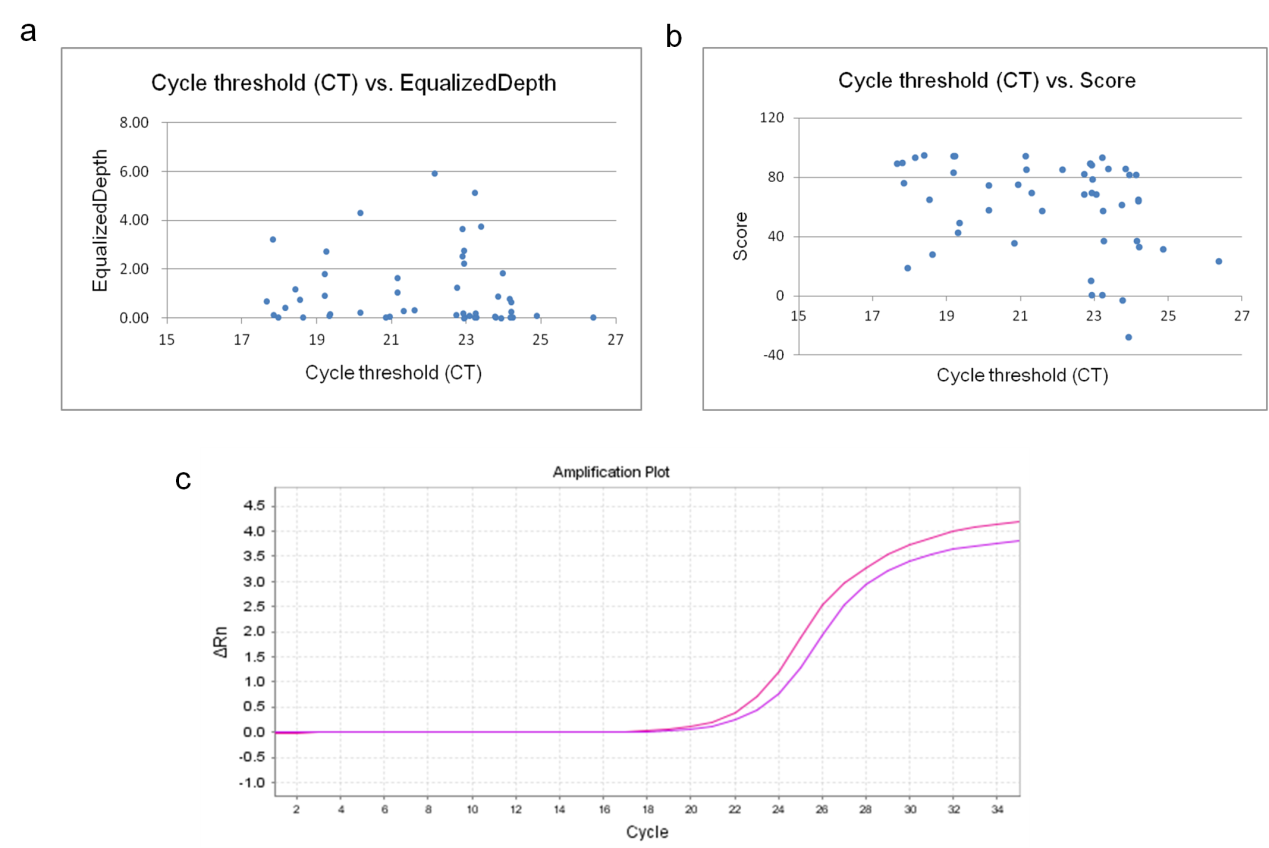

Supplement: Supplementary file 4 — Supplementary Information 4. [file 41598_2022_25561_MOESM4_ESM.docx]
